# Gene sequence analysis of toxins from the spider *Phoneutria nigriventer* revealed an intronless feature

**DOI:** 10.1590/1678-9199-JVATITD-2019-0075

**Published:** 2020-04-30

**Authors:** Ana Luiza Bittencourt Paiva, Alessandra Matavel, Bruno César Souza Silva, Clara Guerra-Duarte, Marcelo Ribeiro Vasconcelos Diniz

**Affiliations:** 1 Diretoria de Pesquisa e Desenvolvimento, Fundação Ezequiel Dias (FUNED), Belo Horizonte, MG, Brazil.; 2 Universidade Federal de Minas Gerais (UFMG), Programa Interunidades de Pós-graduação em Bioinformática (ICB), Belo Horizonte, MG, Brazil.

**Keywords:** Phoneutria, Spider toxins, Toxin genes

## Abstract

**Background:**

*Phoneutria nigriventer* spider venom contains several cysteine-rich peptide toxins that act on different ion channels. Despite extensive studies on its venom and description of cDNA sequences of several of its toxin precursors, the gene structure of these toxins remains unknown.

**Methods:**

Genomic regions encoding the precursors of three previously characterized *P. nigriventer* toxins - PnTx1, PnTx2-5 and PnTx4(5-5) - were amplified by PCR using specific primers. PCR fragments were cloned and sequenced. Obtained sequences were compared with their corresponding cDNA sequences.

**Results:**

The size of PCR fragments obtained and sequences corresponding to genomic regions encoding for the toxin precursors matched their cDNA sequences.

**Conclusions:**

Despite a few nucleotide substitutions in the genomic regions encoding for the toxin precursors when compared with cDNA sequences, the results of the present work indicate that *P. nigriventer* toxins do not contain introns in their genes sequences.

## Background

*Phoneutria nigriventer* is one of the largest existing spiders from the suborder Araneomorphae (RTA clade; family Ctenidae) and one of the few in the world that can cause harm to humans [1,2]. They are wandering, solitary and aggressive spiders, relying on strength and venom toxicity for defense and prey capture rather than using silk webs. *P. nigriventer* venom contains several neurotoxic peptides that act on ion channels and chemical receptors of vertebrates and invertebrates [3].

Although this venom has been studied for over 40 years and cDNA sequences of several toxin precursors have been obtained [4-10], up to this moment there is no investigation on the genome or even gene structure of any of its toxins. 

The earliest spiders dates back to about 300 million years ago. Due to this long existence, together with the high species diversity in this group, spiders have been studied as an interesting evolution model. As an important trait for their evolutionary success, the study of venom and toxin evolution in the molecular level may contribute in elucidating the complex history of spiders [11]. Morphological and behavioral data have been traditionally used to infer phylogeny, but genomic and transcriptomic molecular data are recently challenging previous assumptions on the tree of life of spiders [12,13]. The genetic architecture of their toxin genes has shown to be variable in different spider major groups - such as Araneomorphs [14] and Mygalomorphs [15-18] -, and genes encoding for venom toxins have been described both with and without introns. Therefore, the description of *Phoneutria* toxins gene structure, as a member of the RTA clade (the most diverse group within spiders), is a relevant contribution to the field. 

Several novel components of high molecular mass have been described in *P. nigriventer* venom, but cysteine-rich peptide toxins are by far its most abundant component [4]. PnTx1 (μ-ctenitoxin-Pn1a), PnTx2-5 (δ-ctenitoxin-Pn2c) and PnTx4(5-5) (ɣ-ctenitoxin-Pn1a) are among the best characterized and most abundant cysteine-rich peptide toxins in *P. nigriventer* venom. PnTx1 was demonstrated to inhibit sodium channel currents [19,20] and has shown great neurotoxicity, inducing tail elevation, excitation, salivation, spastic paralysis and lethality in mice [21]. PnTx2-5 acts on sodium channels as well, being able to inhibit sodium channel inactivation [22]. It is also one of the *P. nigriventer*’s most toxic venom components to mice [23] and can induce penile erection, hypersalivation and death by respiratory distress or pulmonary edema [24]. Unlike these two first toxins, PnTx4(5-5) is toxic only to insects [25], showing a remarkable effect on insect sodium channels inactivation [26]. This toxin also seems to hold a biotechnological potential use as a neuroprotective [27] and analgesic drug lead [28]. 

Considering the relevance of *P. nigriventer* toxins and due to their biotechnological, medical and evolutionary importance, we have investigated the gene structure of these three sodium channel modulators toxins from *P. nigriventer* venom, searching for the presence or absence of introns.

## Methods

Venom glands and genomic DNA (gDNA) were obtained from *P. nigriventer* adult spiders maintained at Ezequiel Dias Foundation in Belo Horizonte, Brazil (Sisgen #A26E945). 

### Isolation of RNA and cDNA synthesis

Total RNA was extracted from pooled venom glands of five adult specimens using TRIzol reagent (Invitrogen, USA) according to the manufacturer’s protocol. Subsequently, total RNA was used to synthesize cDNA first strand using Super Script First-Strand Synthesis System for RT-PCR kit (Invitrogen, USA), following the manufacturer’s protocol.

### Isolation of genomic DNA

Genomic DNA (gDNA) was extracted from the leg muscular tissue of an adult *P. nigriventer* specimen, as described by Fan and Gulley [29]. After spider euthanasia, two legs were isolated from the base of the cephalothorax and ground with a chilled, sterile mortar and pestle in liquid nitrogen. After pulverization, the tissue was added to 450 µL of extraction buffer (0.01 M NaCl, 20 mM Tris- HCl pH 8, 1 mM EDTA, 1% SDS and 300 µg/mL proteinase K) and incubated at 55°C for 3h. One volume of phenol:chloroform:isoamyl alcohol (25:24:1) was added and the aqueous phase was collected after centrifugation. DNA was recovered by adding 50 µL of 3 M NaOAc pH 5.2, followed by ethanol precipitation and re-suspended in 100 µL of TE buffer (Tris 10mM, EDTA 1mM). 

### Amplification and sequencing of toxin genes

Sequences of gDNA and cDNA encoding for toxins were amplified by PCR using specific primers (Table 1). Primers sequences for PnTx1 and PnTx2-5 precursors were designed using information from cDNA sequences deposited in GenBank database (accession numbers X73155.1 and AF014463.1, respectively). Primers for PnTx4(5-5) precursor and mature sequences were designed using a cDNA sequence obtained from a *P. nigriventer* venom glands transcriptome study [4]. 

**Table 1.** Primer sequences used for amplification of gDNA and cDNA sequences of each toxin.

Toxin	Primer sequence 5’- 3’
PnTx1	Fw: ATGAAACTTCTGGGGATATTTCTG Rv: GCGAACAAAATCTGACAGC
PnTx2-5	Fw: ATGAAAGTTGCAATCCTCTTC Rv: TCGTGGTATACATAAATCCATATC
PnTx4(5-5)	Fw: ATGAAGGTTGCAATCGTGTT(precursor) TGCGCCGATATTAACGGTGC (mature) Rv: CAGGAATTATGTATTCATGCTGG

PCR reactions contained 100 ng of gDNA or cDNA, 1x PCR buffer, 1 μM dNTPs, 1 μM of each primer and 1 U Platinum Taq DNA polymerase High Fidelity (Invitrogen, USA). Cycling conditions were: 5 min at 94ºC followed by 35 cycles at 94°C for 45 s, 50°C for 45 s, 72°C for 1 min, and a final cycle of 72ºC for 7 min. PCR products were subjected to electrophoresis on 2% agarose gel. gDNA fragments extracted from the agarose gel were purified using Illustra GFX PCR DNA and Gel Band Purification kit (GE Healthcare, USA) and cloned into a pGEM-T Easy vector (Promega, USA). Positive clones were sequenced in an ABI PRISMTM 3700 DNA Automatic Sequencer using the standard M13 reverse primer and Big Dye Terminator v3.1 Cycle sequencing kit (Applied Biosystems, USA).

### Analysis of intron presence in cysteine-rich peptide neurotoxins in available spider genomes

Sequenced spider genomes available in NCBI Genome public database (accession number for each species are as follows: *Loxosceles reclusa* - JJRW01; *Stegodyphus mimosarum* - AZAQ01; *Araneous ventricosus* - BGPR01; *Dysdera sylvatica* - QLNU01; *Trichonephila clavipes* - MWRG01; *Acanthoscurria geniculate* - AZMS01; *Pardosa pseudoannulata* - SBLA01) were used to search for cysteine-rich peptide toxins sequences and evaluate the presence of introns within their genes. 

Since it was not possible to find a specific cysteine-rich peptide toxin sequence published for each of these spiders species, multi FASTA archives were built with known toxin sequences from other spider species, annotated as inhibitor cysteine knot (ICK), knottin or cysteine-rich peptide toxin retrieved from ArachnoServer [30], Knottin database [31] and GenBank [32] to be used as queries to interrogate the genomes. Genomes were used individually as databases to run BLASTn and tBLASTn [33], with default parameters. Results were manually inspected, considering hits with e-value < 1e-05, searching for breaks in the obtained alignment, which would indicate the discontinuity of the sequence, pointing to the presence of introns.

## Results

Using information from cDNA sequences, we designed specific primers in order to amplify genomic regions encoding for the precursors of three well characterized toxins from the spider *P. nigriventer*, namely PnTx1, PnTx2-5, and PnTx4(5-5). The forward primers are complementary to the beginning of the sequence region encoding for the toxins signal peptides. Reverse primers were designed to anneal to the 3’ UTR regions. 

**Figure 1.**
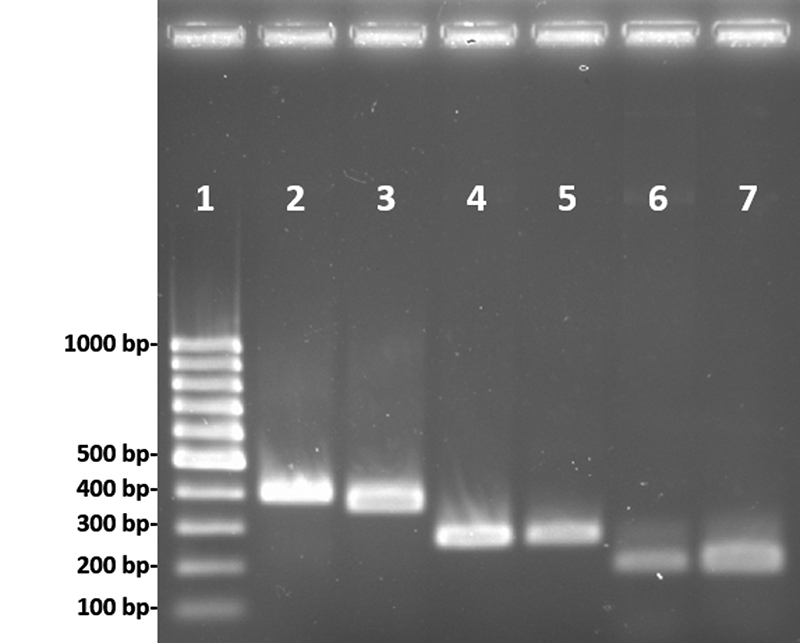
Agarose 2% gel showing the comparison between the fragments of amplified gDNA and cDNA sequences encoding for the toxins. 1: 100 bp; 2: PnTx1 gDNA; 3: PnTx1 cDNA; 4: PnTx2-5 gDNA; 5: PnTx2-5 cDNA; 6: PnTx4 (5-5) gDNA mature sequence; 7: PnTx4(5-5) cDNA mature sequence.

The results showed that the size of PCR fragments corresponding to amplified gDNA encoding for precursor sequences of PnTx1 and PnTx2-5 matched the amplified cDNA fragments, which were expected to be 396 bp and 271 bp, respectively (Figure 1). This indicates that these two toxins do not contain introns in their precursor gene sequences. 

Despite several attempts, we could not obtain any amplification for PnTx4(5-5) using the forward primers annealing to the sequence region encoding for the toxin signal peptide with gDNA as a template. This lack of amplification may be due to the existence of extensive nucleotide variation in DNA sequences encoding for toxins in spiders [34]. Indeed, it has been demonstrated that PnTx4(5-5) is one of the most expressed toxins in *P. nigriventer* venom glands, presenting several isoforms [4]. Thus, we have designed a new forward primer to amplify only the mature sequence of PnTx4(5-5). With this new approach, amplification was observed (Fig. 1) and as for the other tested toxins, gDNA and cDNA amplified fragments presented the same size (181 bp). This indicates that at least the gDNA region encoding for PnTx4(5-5) mature sequence is also intronless. 

**Figure 2.**
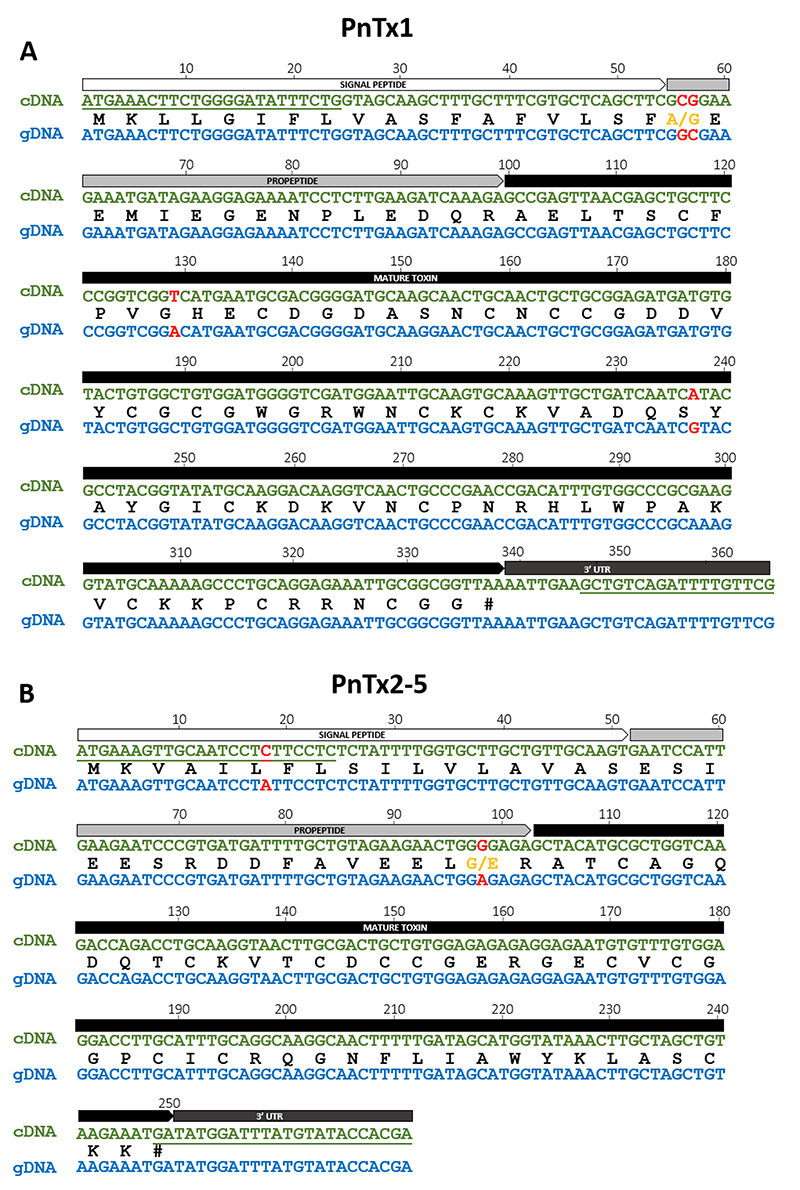
Alignment of cDNA and gDNA sequences of **(A)** PnTx-1 and **(B)** PnTx2-5. The regions corresponding to signal peptide, propeptide, mature toxin and 3’ ÚTR are schematically shown above the sequences in white, gray, black and dark gray boxes, respectively. Primer regions are underlined in cDNA sequences. Nucleotide substitutions are indicated in red letters. The translated protein sequences are shown in black letters between the cDNA and gDNA nucleotide sequences and amino acid substitutions are indicated in orange letters. Hashtag indicates stop codons. cDNA sequences for PnTx-1 and PnTx2-5 were obtained from GenBank accession numbers X73155.1 and AF014463.1, respectively.

In order to confirm if the amplified gDNA sequences of PnTx1 and PnTx2-5 matched the cDNA sequences deposited in the GenBank database (accession numbers X73155.1 and AF014463.1, respectively), the PCR products from gDNA were cloned and sequenced. Each sequence obtained for PnTx1 and PnTx2-5 presented one non-synonymous nucleotide substitution in the propeptide region, leading to one amino acid alteration when compared with the corresponding cDNA sequence deposited in GenBank (Figure 2). Furthermore, PnTx1 gDNA sequence also presented two synonymous substitutions in the toxin mature sequence (Fig. 2A) and PnTx2-5 presented one synonymous substitution in the signal peptide sequence (Fig. 2B). The gDNA sequences obtained for PnTx1 and PnTx2-5 were deposited in GenBank database under the accession numbers MN851275 and MN851276, respectively.

**Figure 3.**
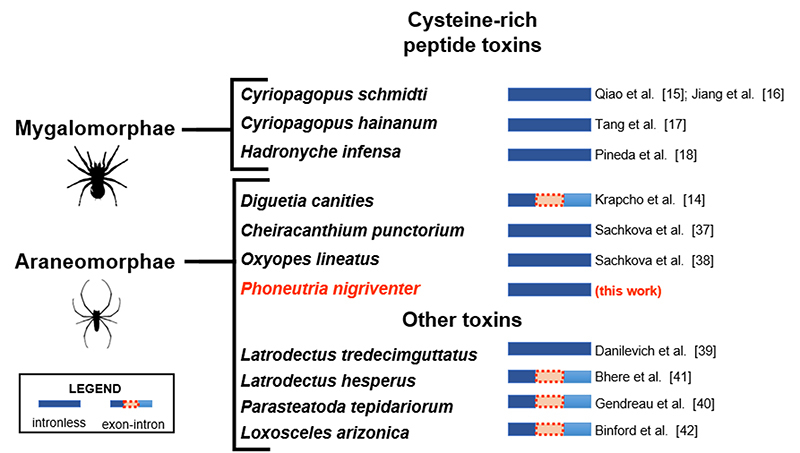
Summary of the presence of introns in spider toxin genes. According to the reviewed literature, all toxin genes from Mygalomorphae spiders studied until now are intronless. In the Araneomorphae group, toxin genes are structured both with and without introns.

Our results were compared with other published data concerning the presence of introns in spider cysteine-rich peptide toxins (Figure 3). Available spider genome sequences were also used for an *in-silico* analysis, using a multiFASTA archive containing several cysteine-rich peptide toxin sequences. We did not find evidence of introns in the retrieved alignments with the genomic sequences of *A. geniculata* (Mygalomorphae*)*, since all alignments were contiguous with the queries. Although most toxin sequences found in *Araneomaphae* genomes seem to be contiguous, at least three sequences in *A. ventricosus,* two sequences in *P. pseudoannulata,* two sequences in *S. mimosarum* and two sequences in *T. clavipes* showed evidence of possible introns, but a more in-depth analysis is required to confirm this.

## Discussion

Spiders are an evolutionary successful group, with a high number of species adapted to different environments. This diversity of adaptations relies on essential molecules produced by them, which compose venom and silk. Studying the gene structure of these molecules may help to elucidate how spiders have evolved and adapted, disclosing possible mechanisms for generating molecular diversity [35]. 

In the present work we have compared the cDNA sequences (derived from mRNA expressed in *P. nigriventer* venom glands), and the genomic sequences (retrieved from muscular tissue of the spiders’ legs) of three of the main toxins from *P. nigriventer* venom, in order to elucidate this Ctenidae toxin gene structure. By comparing cDNA and gDNA sequences of toxins PnTx1 and PnTx2-5, both synonymous and non-synonymous point mutations leading to amino acid substitutions were found in the signal peptide, propeptide and in the mature toxin sequence. These nucleotide and amino acid substitutions were expected since spider toxins are known to have many isoforms, forming groups of related sequences differing by point mutations, even within a single spider specimen, so-called combinatorial libraries [34]. In *P. nigriventer*, the existence of extensive variation in signal peptide, propeptide and mature sequences for toxins was also already demonstrated [4]. In addition, these are abundant toxins that are transcribed at a high frequency, which has been associated with elevated mutation rates, so called transcription-associated mutation (TAM) [36].

The most important finding of the present work is the description, for the first time, of the intronless genomic structure of some important *P. nigriventer* cysteine-rich peptide toxins. Regarding the presence of introns in toxins genes, 11 spider species have been analyzed to date (including this study) (Fig. 3). All spider genes encoding for cysteine-rich peptide toxins from infra-order Mygalomorphae analyzed so far are intronless [15-18]. Among the Araneomorphae*,* the gene for a cysteine-rich peptide toxin found in *Diguetia canities* venom, µ-diguetoxin-Dc1a, has an intron-exon structure [14] whereas two-domain neurotoxins from *Cheiracanthium punctorium* and *Oxyopes lineatus* do not present introns in their toxin gene sequences [37,38], like we have shown in the present report for *P. nigriventer* toxin genes (Fig. 3). 

The analysis of other toxin classes in other Araneomorphae species showed a diverse scenario. Latrotoxins genes were found to be intronless in *Latrodectus tridemciguttatus* [39]*,* but introns were present in these type of toxins genes in *Parasteatoda tepidariorum* [40] and *Latrodectus hesperus* [41]. Sphingomyelinases D from *Loxosceles arizonica* [42] also presented introns in its genes.

To the best of our knowledge, only seven spider genomes have been published to date: *Acanthoscurria geniculata* (Mygalomorphae) and *Stegodyphus mimosarum* (Araneomorphae) [43]; *Trichonephila clavipes* (Araneomorphae) [44]; *Parasteatoda tepidariorum* (Araneomorphae) [45]; *Pardosa pseudoannulata* (Araneomorpha) [46]; *Araneus ventricosus* (Araneomorphae) [47] and *Dysdera silvatica* (Araneomorphae) [48]. In addition, the 5000 arthropod genome initiative (i5K), which is committed to sequencing and analyzing 5000 high-priority arthropods [49], made available a draft of *Loxosceles reclusa* genome. Introns were described as present in all these spider genome analysis, but whether introns are present or not specifically in cystein-rich peptide toxin genes was not informed. As previously mentioned, latrotoxins found in the genome of the common house spider *P. tepidariorum* did present introns in their gene structure [40]. 

To increase the body of information regarding the presence of introns in cysteine-rich peptide toxins, we attempted to use the available spider genomic data to analyze the structure of cysteine-rich peptides in toxin gene sequences. We did not find evidence of introns in the genomic sequences of *A. geniculata* (Mygalomorphae), corroborating the previous findings in the literature for this group. Regarding Araneomaphae spiders, the *in-silico* preliminary analysis did not show evidence of intron presence in cysteine-rich peptide toxins in two species (*L. reclusa* and *D. sylvatica).* However, despite most of the toxin gene sequences being apparently intronless, in the other four analyzed species (*A. ventricosus, P. pseudoannulata, S. mimosarum* and *T. clavipes)* the discontinuity of some alignments pointed to the possible presence of introns in a few toxins gene sequences. 

Here, our experimental results showed that *P. nigriventer* do not present introns in the cysteine-rich peptide toxin sequences analyzed, corroborating most of the other experimental and *in-silico* findings that indicate the absence of introns in this class of spider toxins. However, since in the genomic data analysis we found evidence of the occurrence of introns in some toxin gene sequences from other Araneomorphae spiders, we cannot discard the possibility that other *Phoneutria* toxins can also present introns in their sequences. For instance, the co-existence of gene copies both with and without introns has been demonstrated for the elongation factor-1α (EF-1α) in Salticidae spiders from the genus *Habbronatus* [50]. 

Pineda et al. [18], in their work describing the gene structure of toxins from Australian funnel-web spiders, hypothesized that Mygalomorphae spiders had lost their toxin gene introns through their evolutionary history, whereas Araneomorphae kept them from a common ancestor. However, considering that different families of toxins have different features concerning the presence of introns and no consensus could be found among Araneomorphae, the evolutionary history of spider peptide neurotoxins seems to be more complex and remains to be further clarified.

Venom toxins are essential for spider survival and a high level of toxin expression in the venom glands is required. Gene expression increase may occur through the polyploidization of venom gland tissue cells [51]. Intron loss is another possible mechanism for increasing gene expression, since it has been reported that highly expressed genes tend to lose introns more frequently and gain introns more rarely than genes with low expression levels [52]. Furthermore, the absence of introns has also been related to an increase of mutation rates [53], which in turn may contribute to the high variability of toxin sequences and the emergence of new spider toxins [38]. On the other hand, the presence of introns in toxin genes may modulate the mutation rates of each exon separated by them [54], constituting a different mechanism for toxin evolution and diversification. Besides that, as most of spider toxin DNA sequences come from venom gland transcriptome analysis , the roles of alternative splicing, gene duplication and other regulatory controls in generating venom molecular diversity still need to be further studied.

## Conclusions

In this work, we have investigated the structure of the genes encoding for three known sodium channel modulator toxins from *P. nigriventer* venom and the results indicate that *P. nigriventer* toxins do not contain introns in their genes sequences. However, since we also found evidence of toxin genes with and without introns in genomes of other Araneomorphae spiders, we cannot rule out the possibility of the presence of introns in other *P. nigriventer* toxin genes. This can only be confirmed after sequencing its genome.

As the majority of spider toxin DNA sequences come from venom glands transcriptomes studies, the gene structure of toxins from most spider toxins are still obscure. Thus, we believe that our results can contribute to future studies on understanding the mechanisms underlying spider venom molecular diversity, as well as the evolutionary aspects of spider toxins. 

### Abbreviations

gDNA: genomic DNA; ICK: inhibitor cysteine knot; PnTx1: μ-ctenitoxin-Pn1a; PnTx2-5: δ-ctenitoxin-Pn2c; PnTx4(5-5): ɣ-ctenitoxin-Pn1a; TAM: transcription-associated mutation.
